# Supplementary material for: Reliable retrieval is intrinsically rewarding: Recency, item difficulty, study session memory, and subjective confidence predict satisfaction in word-pair recall
Source: PLoS One. 2023 Oct 19;18(10):e0292866. doi: 10.1371/journal.pone.0292866 (PMC10586604; doi:10.1371/journal.pone.0292866)
Supplement: Attachment — Submitted filename: Review_PlosOne_Memory and satisfaction.pdf [file pone.0292866.s001.pdf]

PONE-D-23-11730 - Review

Summary

The authors present a study in which they assess whether recall is rewarding. They set out to investigate two mutually exclusive accounts: either distant, more difficult-to-retrieve memories would produce a stronger sense of satisfaction (internal reward) when recall is successful or, alternatively, recent, easier-to-retrieval memories would produce a stronger sense of satisfaction because they do not require great effort. The authors find evidence for the latter account.

The manuscript is well-written, and the findings are very interesting. There are a few points, however, that require more elaboration, particularly in the Results section, before we can recommend the text for publication. Also, we suggest that the authors share, via OSF, all the analysis files, so that others readers can reproduce the results from the data already shared. Below we elaborate on some major and minor points.

Major points

1. In the title and throughout the manuscript, the use of the term *reliable* and its variants (e.g., "reliable retrieval") seemed misleading to me. I understand that *reliability*, in memory research, refers to the accuracy or the "truthfulness" of the recalled information according to some external objective criterion (e.g., in eyewitness testimony research). Reliability may also have very specific meanings in psychology, such as *test-retest reliability*. However, in this manuscript, the term reliability was used as synonymous with *confidence* (e.g., "recall confidence"), which refers to the degree of subjective certainty learners assign to their response (i.e., the subjective confidence in their own memory). The interchangeable use of the terms *reliability* and *confidence* may inadvertently confuse the reader. To improve readability and keep consistency with the memory literature in general, I suggest replacing the term *reliability* and its variants with *confidence* and its variants (e.g., confident retrieval or memory confidence) when the aim is to refer to learners' subjective judgments.

2. In the Abstract, the authors make the following statement (brackets added by me): "We found that retrieval satisfaction decreased with time since encoding and [also decreased with] session retrieval performance." This statement seems to be based on results presented (lines 281–285), showing a Confidence × Rasch Performance interaction. However, in the Results (lines 328–329), the authors claim that "… For satisfaction, we found that session performance $b_{session\ perf.} = 0.50$, $t = 3.35$, $p < .001$ and $LR = 9.19$, $p <.01$." The term "session [retrieval] performance" seems to imply two different meanings throughout the manuscript. In Rasch analyses, it seems to refer to the *estimated* item-by-participant performance in the first retrieval attempt across the encoding sessions (based on items and participants parameters estimated with the Rasch model), a proxy of pre-retrieval uncertainty, as stated by the authors (one estimate per participant). In final-recall test analyses, however, "session performance" seems to refer to the *observed* final-recall performance (by session; five estimates per participant), used to assess whether the empirical final recall as a function of different retention intervals affect retrieval satisfaction and retrieval confidence. These two senses of "session performance" seem to show different relationships with satisfaction. To improve readability, I strongly recommend that the authors make this distinction

explicit and, in the Discussion section, discuss the implications of each of these results for the hypotheses presented.

3. Lines 217–220: "Upon pressing yes, the user was presented with text explaining a time gap before the answer was presented. After a 5 s waiting time elapsed, the Swahili word that they wished to know the English translation to was presented on the left, and the translation on the right of a crosshair in the center of the screen".

- Why did the authors insert a 5-s delay until the presentation of the correct English word? This introduces a "cost of response", rendering the response "Yes, I am curious" more time consuming than "No, I am not curious". Given the context of a long, multisession experiment, in which participants are already tired, introducing such 5-s cost is likely to reduce the number of "Yes" responses. Therefore, we suggest that the authors explain why they have chosen to include the 5-s delay and how it could have affected the results and conclusions of their study.

4. Lines 231–232: "Participants recalled 61% of the items in their first recall attempt and never needed more than five repetitions until they correctly recalled an item". One characteristic of the adopted within-subjects design is that it confounds the time since encoding with the number of intervening lists until the final test (which, speculatively, may have led to a buildup of proactive interference). Did the initial recall reported in the text differ across the five sessions? Please explicitly state this information in the text.

5. Lines 232–235: The authors compared satisfaction and confidence between correct and incorrect retrieval attempts. Among the incorrect attempts, what was the rate of response omissions? If the omission rate was high, I believe it is important to restrict the analyses only to incorrect attempts *with responses*, as it would not be surprising for satisfaction and confidence to be higher in correct attempts if the incorrect attempts include many omissions.

6. Lines 247–248: "As seen in figure 1, participants' retrieval confidence and satisfaction increased between first and final retrieval attempt". Given the dropout procedure adopted in the study, I wonder how the authors defined "final retrieval attempt." For example, if for a given participant, the last test repetition led to correct recall of the remaining five items, did only those five items contribute to the measures of confidence and satisfaction? Or, alternatively, did the final attempt of *each item* contribute to the measures of confidence and satisfaction? Could you explain this point?

7. The sample size (n = 30) is quite small for estimating Rasch models. Some points:

a) Please discuss the consequences of the small sample size on the Rasch model estimates (e.g., how would that impact on the relationship between item difficulty and satisfaction)?

b) Rasch model estimates were used as indices of item difficulty. Nelson and Dunlosky (1994) provide norms of recall difficulty for a sample of 100 Swahili-English word pairs. Why did the authors decide to estimate item difficulty from Rasch models rather than from Nelson and Dunlosky's norms? As a suggestion, the authors could correlate their Rasch model estimates with Nelson and Dunlosky's (1994) norm scores as a means of validating the items' difficulty estimates.

c) Additionally, the authors could correlate item difficulty estimates from Figure 2 to the proportion recalled in the final test from Figure 3 (left panel) as an attempt to validate independently their estimates of the Rasch models.

8. Model comparisons (part 1). The excerpts below refer to lines 267–285. In this section of the Results, the authors describe a series of mixed-effect model comparisons to support the patterns described in the figures. The analyses here (part 1) focus on the fixed-effect terms *Rasch performance* (item difficulty) and *Confidence* and their interaction. For ease of reference, we will use numbered equations to refer to each mixed-effect linear regression model and the text passage it refers to.

The main question we have here is whether the full model, with the fixed effects of Confidence, Rasch performance and their interaction (Equation 6) is significant when compared to simpler models (Equations 3, 4 and 5). If it is, the role of the interaction should be emphasized in the Abstract and in the Discussion, as the result implies that that Satisfaction is influenced by response Confidence, item difficulty and the Confidence × Rasch performance interaction (for a given level of confidence, correct responses to more difficult items are associated with higher satisfaction).

Below we reproduce the models and the corresponding text to elaborate on our issue regarding model comparison. The simplest model assessed was the following:

1. Satisfaction ~ Rasch performance + (Rasch performance | participant)

(1) “The model Satisfaction ~ Rasch performance + Rasch performance | participant yielded b_{Rasch} = 0.35, t = 2.73, p < .01 with 1820 observations. The model with Rasch performance as a fixed effects term was reliably better supported than a null model without the fixed effects term, LR_{fixed} = 8.0, p < .01.” Thus, participants’ satisfaction with their own answers was predicted by item difficulty. The model with the Rasch performance term was compared to the model without that term. The authors also assessed whether confidence could be predicted from Rasch performance. It did:

2. Confidence ~ Rasch performance + (Rasch performance | participant)

(2) “We tested the corresponding effect replacing satisfaction with confidence and found that b_{Rasch} = .40, t = 3.2, p < .01. The model with Rasch performance as fixed effects term was reliably better supported than a null model without the term, LR = 10.0, p < .001.” Next, the authors assessed Satisfaction as function of Confidence:

3. Satisfaction ~ Confidence + (Rasch performance | participant)

(3) “We also tested response confidence as a predictor of satisfaction by running confidence ratings as a fixed effects term in a linear mixed effects model and found a reliable association between satisfaction and confidence ratings with b1 = 0.72, t = 17.0, p < .001. The results indicated that the easier the item is to recall, the more satisfying is the experience.” Here, the last sentence refers to item difficulty, when it should refer to Confidence, as Confidence was the fixed-effect term assessed in Equation 3. The authors then proceed to assess whether the interaction term is significant:

4. Satisfaction ~ Confidence × Rasch performance + (Rasch performance | participant)

(4) “However, the possibility still exist that ease recall and confidence may interact in satisfaction such that items that appeared difficult to recall (as indicated by the Rasch performance estimate) yet were confidently recalled, appeared as more satisfying than

an equally confident recall of an item that was easier to retrieve. We therefore tested the model Satisfaction ~ Confidence x Rasch performance + Rasch performance | participant. Indeed, the interaction was reliable negative at $b_{Confidence\ x\ Rasch} = -.25$, $t = -2.83$, $p < .01$, the likelihood ratio for the interaction model over the null model was LR = 1266, $p < .01$ suggesting that for equal confidence, more difficult items (i.e., low Rasch performance) induced higher satisfaction ratings when successfully recalled." Here the authors report a significant Confidence × Rasch performance interaction and describe what it means. However, the interaction model (Equation 4) is compared against a null model without the interaction term and without fixed-effect terms. Our contention is that to assess the role of the interaction, a full model (Equation 6) should be compared with a simpler model with the other fixed-effect terms and without the interaction term (Equation 5).

5. Satisfaction ~ Confidence + Rasch performance + (Rasch performance | participant)

6. Satisfaction ~ Confidence + Rasch performance + Confidence × Rasch performance + (Rasch performance | participant)

If this model comparison proves significant, then the Results, Discussion and Abstract should emphasize the interaction, as it is potentially a novel and interesting finding. Unlike argued by the authors in the Discussion, lines 364-373, successful recall of more difficult items, for a given confidence level, would be associated with higher levels of satisfaction (a result consistent with the "desirable difficulties" framework).

9. Model comparisons (part 2). The excerpts below refer to lines 319–330 in the manuscript. In this passage of the Results, the authors also describe a series of mixed-effect model comparisons. The analyses here (part 2) focus on the fixed-effect terms *log(time)* (time since encoding) and *Session performance* (participant session performance average recall). For ease of reference, we will use numbered equations to refer to each mixed-effect linear regression model and the text passage it refers to.

(1) "Furthermore, we employed a mixed effects linear regression test of retrieval satisfaction for correctly recalled items as a function of log time since encoding as fixed effect and with participant as random variable. With 1172 observations, the fixed effect of log time since encoding was $b_{log(time)} = -0.027$, $t = -2.19$, $p < .05$, with LR = 9.19, $p < .001$."

1. Satisfaction ~ log(time) + (1 | participant)

(2) "The corresponding analysis with time since encoding as a predictor of confidence rating yielded $b_{log(time)} = -0.03$, $t = -2.73$, $p < .01$ with LR = 6.30, $p < .05$."

2. Confidence ~ log(time) + (1 | participant)

(3) "Finally, noticing that recall rates varied substantially across sessions and between participants (see left panel of figure 3), we tested participant session performance average recall score as a predictor of correct recall satisfaction and response confidence, respectively. For satisfaction, we found that session performance $b_{session\ perf.} = 0.50$, $t = 3.35$, $p < .001$ and LR = 9.19, $p <.01$."

3. Satisfaction ~ Session performance + (1 | participant)

(4) "For confidence ratings, session average performance $b_{session\ perf.} = 0.59$, $t = 3.88$, $p < .0001$ and LR = 12.0, $p < .0001$."

4. Confidence ~ Session performance + (1 | participant)

Here we ask why the authors did not follow a sequence of model comparisons similar to the ones carried out for *Confidence* and *Rasch performance* (i.e., model comparisons with progressively more complex models). In particular, we ask if the authors tested whether Satisfaction is affected by a log(time) × Rasch performance interaction. This interaction would be of considerable theoretical interest because both log(time) and Rasch performance are terms related to item difficulty. It thus stands to reason that the Satisfaction for the most difficult combination would be very different from Satisfaction for the least difficult combination (most difficult combination: high log(time) and low Rasch performance; least difficult combination: low log(time) and high Rasch performance). A model comparison between models 5 and 6 below would address this question.

5. Satisfaction ~ log(time) + Rasch performance + (1 | participant)

6. Satisfaction ~ log(time) + Rasch performance + log(time) × Rasch performance + (1 | participant)

10. Please include on the OSF site the analyses' files for the Rasch models and the mixed-effects linear regressions. Rasch models are not common in the memory literature, and so it would be very helpful for other researchers to have access to an implementation of this type of analysis. In addition, mixed-effect analyses may become complicated to follow from text only (as opposed to looking at the analysis code). Thus, we also ask the authors to make the mixed-effects linear regression code available.

Minor points

-Line 141: "Out of 70 participants who completed the first session a total of 30 participants (…) completed all sessions…". Attrition rate was very high (> 50%). At a minimum, the authors should present a summary of the main characteristics of this dropout sample (mean age, dropout pattern – e.g., most dropped after the first session, followed by second session and son on?). This could provide a more complete picture for the reader as to what kind of participant remained in the studied (e.g., unusually motivated students with higher levels of intrinsic curiosity). For example, one could argued that the relationship between satisfaction and confidence observed in the study, may suffer from self-selection bias, in that individuals who already find the act of recall rewarding (satisfying) were the one that remained in the final sample of the study.

-Some abbreviations are not in their long form upon first presentation. Examples: Line 171 - GDPR (General Data Protection Regulations), Line 284 - LR (Likelihood ratio)

-The terms "word list" (line 148) vs. "wordlist" (line 178) are used interchangeably in the text. We suggest the use of a single form (e.g., "word list").

-Line 204: Encoding session the 7th day => Encoding session in the 7th day

-Lines 188–192: Small inconsistencies in the response scales. Most scales present a prefix “Un-” on their lowest value, but there is no prefix for “Satisfied” (i.e., “Not Satisfied”). Was there any reason for this choice of words? One possibility would have been “Dissatisfied”, as in (-1 = Dissatisfied, +1 = Satisfied) – same applies to lines 211-212 (-1 = Not Satisfied, +1 = Satisfied).

-Lines 188–192: It was not immediately clear how many levels each slide bar entails (e.g., three values as in [-1, 0 , 1] or five values as in [-1 , -.5, 0, +5, +1]). From the text, I implied that the slide scale is continuous, with values ranging from -1 to 1. It would help to make this more explicit in the text.

-Line 195: “…similarities according to the Levenshtein’s distance algorithm”. Since the use of the Levenshtein’s algorithm is not very common in memory studies, it would be helpful to add a sentence or two giving a short explanation of the algorithm (i.e., how it considers as correct a response in which a typo was mistakenly typed by the participant).

Lines 278: “recall and confidence may interact in satisfaction” => may interact with satisfaction
