# Supplementary material for: Reliable retrieval is intrinsically rewarding: Recency, item difficulty, study session memory, and subjective confidence predict satisfaction in word-pair recall
Source: PLoS One. 2023 Oct 19;18(10):e0292866. doi: 10.1371/journal.pone.0292866 (PMC10586604; doi:10.1371/journal.pone.0292866)
Supplement: Attachment — Submitted filename: Review_R1_PlosOne_Memory and satisfaction.pdf [file pone.0292866.s003.pdf]

PONE-D-23-11730 – Review R1

Summary

The authors have addressed satisfactorily the issues raised in the previous version. Here I would like to highlight minor points concerning clarity and copyediting details before recommending the manuscript for publication.

Clarity

1. In the Figure 1, panels A and B, it is not entirely clear to me what the x- and y-axis represent. Below is a copy of Fig. 1(A) where two data points have been highlighted. My understanding is as follows. Each point represents one participant. The participant in the green square represents someone who had a relatively good initial recall in the first session (e.g., mean correct recall in session 1 = .51) and a perfect recall in the least session (mean correct recall in session 5 = 1.0; I presumed this latter case applied to all cases, since the design followed a drop-out procedure). Thus, the retrieval improvement on the x-axis for the participant in green is 1.0 − .51 = .49. The learning-related confidence change is the difference between the mean confidence for the correct attempts in session 1 and the mean confidence for the correct attempts in session 5.

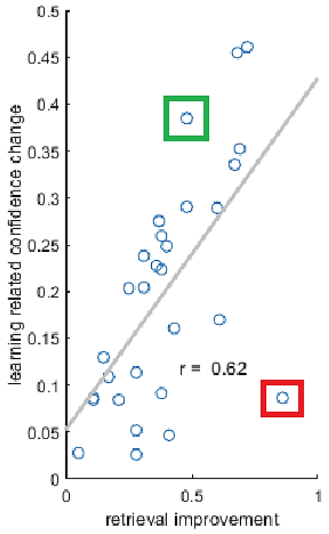

Following the same logic, the retrieval improvement for the participant in the red box is the mean correct recall in session 5 (1.0) minus the mean correct recall in session 1 (e.g., 0.10). Thus, the retrieval improvement for the participant in the red box is high at .90. The corresponding difference in confidence ratings is low at 0.09. Is that reasoning correct? It would be helpful if the authors described in a short paragraph how they obtained one of the points in Fig. 1, panel A.

Minor points

In the following, I present a list of small suggestions to improve readability and compliance with *Plos One* copyediting standards.

-p.2, line 30: “to successfully retrieve” → “to successfully retrieve **it**”.

-p.2, line 36-37: “We found that retrieval satisfaction decreased with time since encoding and study session retrieval performance”. → “We found that retrieval satisfaction decreased with time since encoding and **with lower** study session retrieval performance”.

-p.2, line 38: “But we also found … indicating” → “We also found … **, indicating**”.

-p.5, line 105: “reward at play the presence of” → “reward at play **in** the *presence*”.

-p.11, line 255: The data file in the OSF platform was uploaded with the .txt extension. It would be more helpful if it were uploaded with the the **.csv extension**, as the OSF system would be able to read the file and print it on the screen of the web browser.

-p.12, line 289 (section “Encoding section results” and wherever applicable): The abbreviations for mean (*M*) and standard deviation (*SD*) should be reported in **capital letters and italics**. Please revise all such cases in the manuscript (e.g., p.15, section “Final recall test results”).

-p.13, line 309 (Figure 1 caption): The first letters should be capitalized in the boldface caption: “Study Session Learning and Ratings”. In addition, the legends in the x- and y-axis should have the first letter of each word capitalized: x = Retrieval Improvement; y = Learning-related confidence change. Same applies for panels B and C.

-p.13, line 317: References to Figures should be abbreviated (Figure 1 → **Fig. 1**; apply throughout the text for figure 1 → Fig. 1, figure 2 → Fig. 2, and figure 3 → Fig. 3).

-p.15, line 362 (Figure 2 caption): The first letters are capitalized in the boldface caption: “Performance and Rating Associations”. The legends in the x- and y-axis should have the first letter of each word capitalized: x = Estimated performance (Rasch); y = z(Satisfaction Rating). Same applies for panels B and C.

-p.17, line 413 and line 415: Change the *b* subscripts to keep consistency with new terminology (performance memory → session memory), so that b_{session.perf} becomes **b_{session.mem}**. One case on p. 413, and the other on p. 415.

-p.18, line 436: “z-scored JOL rating at study.” → “z-scored JOL rating **unit** at study.”

-p.20, line 489: “…participants who did not complete the experiment were slightly more likely to be male …”. Shouldn’t it be **female**? On p. 11, line 262, the authors say that “Of those who did not complete all sessions, 20 identified as female and 11 as male”. So, the claim on p.20, line 489 should be “more likely to be female”.

-p.20, line 499: “…, what then might the adaptive value … be?” → “…, what then might **be** the adaptive value …?”

-p.22, line 542: Here I am not sure which preposition is the most appropriate, as it depends on the meaning the authors wish to convey. The text reads “It is also possible that the learning reward argument in curiosity theory is incorrect or at least that the hedonic ‘wanting’ (Berridge & Kringlebcach, 2015) [Note: please use *Plos One* reference style here] aspect of reward is irrelevant **in** curiosity.” Shouldn’t it be “irrelevant **to** curiosity”?
